# Editorial: Advanced cryogenic tools to preserve gametes, embryos, and gonadal tissues

**DOI:** 10.3389/fvets.2023.1267966

**Published:** 2023-09-04

**Authors:** Barbara Merlo, Joanna Maria Gonçalves Souza-Fabjan, Cristina Soriano-Úbeda, Jennifer B. Nagashima

**Affiliations:** ^1^Department of Veterinary Medical Sciences, University of Bologna, Bologna, Italy; ^2^Faculdade de Veterinária, Universidade Federal Fluminense, Niterói, Brazil; ^3^Department of Veterinary Medicine, Surgery, and Anatomy, Universidad de León, León, Spain; ^4^Center for Species Survival, Smithsonian National Zoo and Conservation Biology Institute, Front Royal, VA, United States

**Keywords:** oocyte, sperm, embryo, cryopreservation, freezing, vitrification, lyophilization

The need to advance cryogenic technologies for fertility preservation is becoming increasingly critical across a variety of fields. In humans, these techniques are important not only to address fertility problems, but for men and women with medical concerns or treatments affecting reproductive potential, or those wanting to delay child-bearing years. In species of agricultural value, similar tools can be applied to assist in production efficiency, whereas, in endangered animals, the goal of maintaining genetic diversity in small populations may only be possible through the application of gamete and gonadal tissue cryopreservation. This Research Topic highlights the challenges and areas of advancement in the field of cryopreservation for fertility preservation (Figure 1).

**Figure 1:**
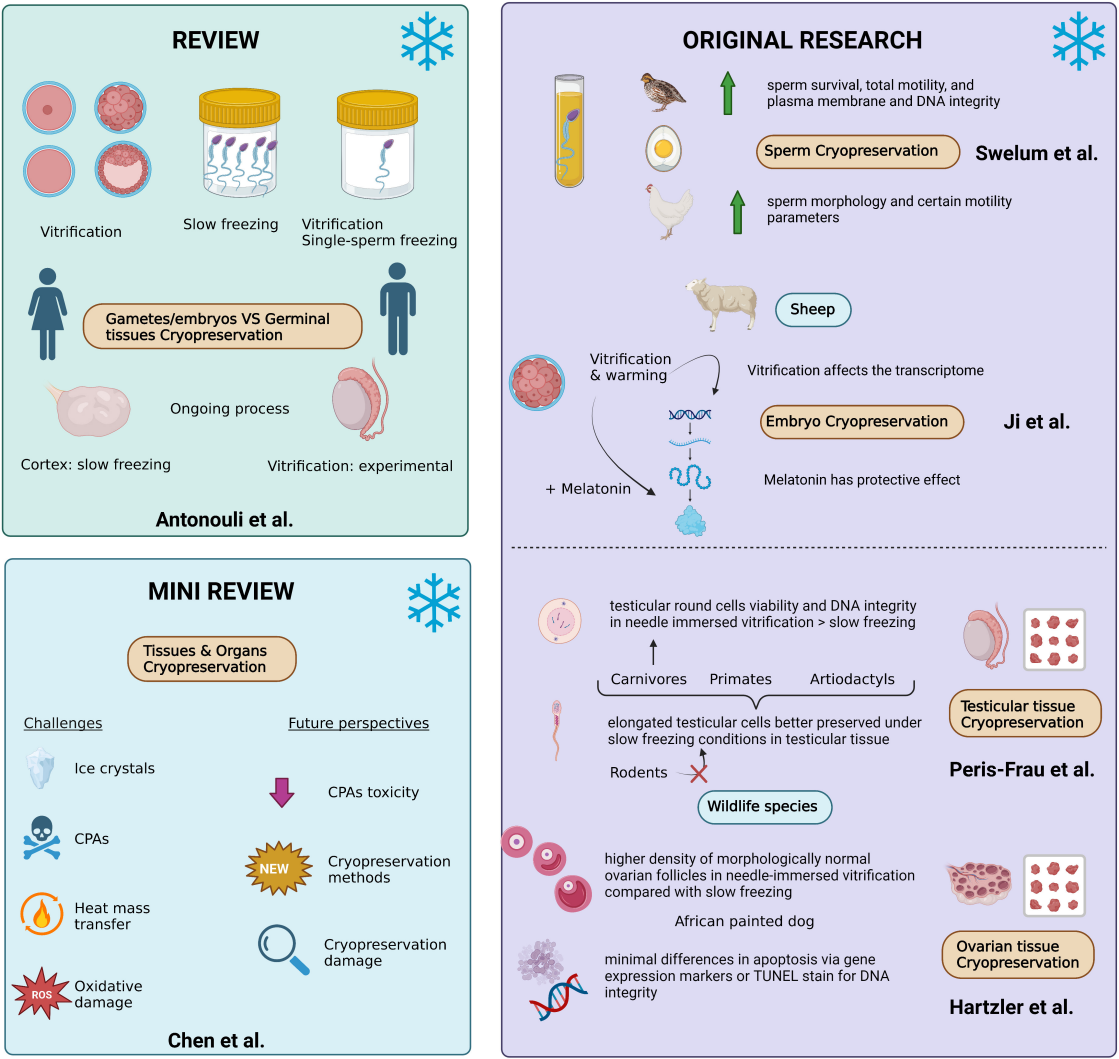
Visualization of the Research Topic “*Advanced Cryogenic Tools To Preserve Gametes, Embryos, And Gonadal Tissues*” (created in Biorender.com).

Currently, the two most commonly utilized methods for preservation using cryogenics are slow freezing, in which cells are progressively cooled to −196°C, either controlled via a programmable freezer, or uncontrolled in a container with an internal cooling rate of ~1°C/min, and vitrification, a rapid process in which the liquid phase of a cell or tissue solidifies into a glassy phase without ice crystal formation. Both techniques are regularly applied in human clinics. In the review paper by Antonouli et al., the cryopreservation protocols most commonly used in human fertility preservation for each cell/tissue type are detailed, including describing the growing preference for vitrification methods for oocyte and *in vitro*-derived embryo cryopreservation, comparing and contrasting recently developed tools for open (direct liquid nitrogen contact) vs. closed vitrification systems, and discussing the concerns for patient accessibility to necessary fertility preservation options and their variable regulation across countries.

Despite their breadth of use in human clinics, no cryopreservation protocol is fully without its challenges. Potential cell damage may occur due to ice formation, cryoprotectant toxicity, and/or thermal, oxidative, and osmotic stress to the cells. Further, cryopreservation conditions which are successful in humans may have very different outcomes when utilized in other species. This includes ram sperm, which are particularly sensitive to cold-shock due to their low membrane cholesterol:phospholipid ratio. In the research by Swelum et al., Japanese quail egg yolk (higher water and select monounsaturated fatty acids and trace elements content) was compared with the gold-standard chicken egg yolk (higher protein and poly-unsaturated fatty acid content) and a mixture of the two for their ability to support ram sperm cryopreservation. They found that the chicken egg yolk-based semen extender maintained the highest levels of sperm survival, total motility, and plasma membrane and DNA integrity, whereas sperm morphology and certain motility parameters were better supported by the quail egg yolk-based extender. These variances were attributed to differences in fatty acid, protein, fat, moisture, and ash content in quail vs. chicken yolks, and represent insight into the relative contributions of the broadly-used but not fully understood egg yolk cryo-diluent.

Focusing on embryos, in the research by Ji et al., the authors assessed the influence of melatonin supplementation on combating the oxidative stress of cryopreservation in sheep morulae. While vitrification significantly altered several gene expression pathways compared with fresh controls, the authors demonstrated that within-group variation was higher than treatment-based variation, suggesting that individual embryos responded differently to cryopreservation. They also found that melatonin supplementation in vitrified ovine morulae upregulated several key embryonic development-associated genes in the transforming growth factor β and forkhead box subfamily pathways, as well as supporting XPA expression, part of the Nucleotide excision repair system for DNA damage, following vitrification.

As reviewed by Chen et al., the technical challenges presented by cryopreservation can be enhanced in tissues and organs, as the samples in question represent heterogeneous cell populations with complex architecture and cell-cell interactions, while also facing the additional task of needing rapid and even heat transfer across a large mass. Despite this, gonadal tissue cryopreservation efforts are rapidly expanding for both human and endangered species work, as options to collect mature gametes are often limited (i.e., in prepubertal children, or in animals outside of the breeding season). The research described by Hartzler et al. and Peris-Frau et al. evaluated which of these methods better supported the survival of gonadal tissues for wildlife species, in ovarian and testicular tissues, respectively. In both studies, slow freezing of gonadal tissues was compared with needle-immersed vitrification, in which tissues are threaded onto a small-gauge needle for cryoprotectant equilibration and plunged directly into liquid nitrogen. Hartzler et al. demonstrated that African painted dog ovarian tissue maintained a higher density of morphologically normal ovarian follicles in needle-immersed vitrification compared with slow freezing, but minimal differences in apoptosis via gene expression markers or TUNEL stain for DNA integrity were observed among cryopreservation groups.

Highlighting the challenges both of cell heterogeneity in organs and species-specific differences in cryo-sensitivity, Peris-Frau et al. determined that carnivore testicular round cells (spermatogonia through to early spermatids) had higher viability and DNA integrity in needle-immersed vitrification-treatments compared with slow freezing. However, most of the 33 species assessed in the study—including artiodactyls, carnivores, and primates—elongated testicular cells (elongated spermatids and spermatozoa) were better preserved under slow freezing conditions in testicular tissue, with only rodents displaying no significant difference among the two cryopreservation treatments. Together, these studies advance our understanding of the species- and tissue-specific needs for gonadal tissue cryopreservation for species conservation. Nevertheless, new advances in low-toxicity cryoprotectants, CPA delivery/perfusion methods, and novel nano-warming techniques hold promise for gonadal tissue cryopreservation efforts, as reviewed by Chen et al.. Moreover, the use of cryopreserved-warmed gonadal tissue either via tissue transplantation or *in vitro* culture reviewed by Antonouli et al. represents growing areas of research to both utilize preserved tissues for fertility restoration, and also to improve our understanding of the mechanisms of cryo-damage such that we may continue to develop tools to address them moving forward.

## Author contributions

BM: Visualization, Writing—review and editing. JS-F: Writing—review and editing. CS-Ú: Writing—review and editing. JN: Writing—original draft.

